# *Lactobacillus murinus* Improved the Bioavailability of Orally Administered Glycyrrhizic Acid in Rats

**DOI:** 10.3389/fmicb.2020.00597

**Published:** 2020-04-24

**Authors:** Tianjie Yuan, Jin Wang, Letian Chen, Jinjun Shan, Liuqing Di

**Affiliations:** ^1^School of Pharmacy, Nanjing University of Chinese Medicine, Nanjing, China; ^2^Jiangsu Engineering Research Centre for Efficient Delivery System of TCM, School of Pharmacy, Nanjing University of Chinese Medicine, Nanjing, China; ^3^Jiangsu Key Laboratory of Pediatric Respiratory Disease, Nanjing University of Chinese Medicine, Nanjing, China

**Keywords:** glycyrrhizic acid, bioavailability, *Lactobacillus murinus*, oral administration, intestinal microbiota

## Abstract

Intestinal microbiota has been extensively studied in the context of host health benefit, and it has recently become clear that the gut microbiota influences drug pharmacokinetics and correspondingly efficacy. Intestinal microbiota dysbiosis is closely related with liver cirrhosis, especially the depletion of *Lactobacillus*. Therefore, the bioavailability of orally administered glycyrrhizic acid (GL) was speculated to be influenced under a pathological state. In the present study, *L. murinus* was isolated and screened for GL bioconversion capacity *in vitro*. Compared with *Lactobacillus rhamnosus* and *Lactobacillus acidophilus*, *L. murinus* was chosen for further investigation because it has the highest biotransformation rate. Our results showed that *L. murinus* could significantly improve the translocation of GL on Caco-2 cell models. Meanwhile, *L. murinus* was observed to have the ability to bind with the surface of Caco-2 cells and prominently downregulate the transporter gene expression level of multidrug resistance gene 1 (MDR1) and multidrug resistance protein 2 (MRP2), which were involved in the efflux of drugs. Furthermore, *L. murinus* was selected to be orally administred into rats in healthy and liver cirrhosis groups by a daily gavage protocol. Our data highlighted that supplements of *L. murinus* significantly improved the bioavailability of orally administered GL in rats, especially under a pathological condition, which may provide a novel strategy for improving the clinical therapeutic effect of liver protective drugs.

## Introduction

The human microbiome is composed of trillions of bacteria, viruses, and fungi, whose collective genome contains at least 100-fold more genes than the complete human genome (Stojancevic et al., 2014). By far, the organ colonized with the largest microbial population is the gastrointestinal (GI) tract, which contains over 70% of all the microbes in the human body (Sekirov et al., 2010). The intestinal microflora that form a complex ecosystem play a key role in the modulation of human health and diseases. Additionally, gut microbiota has also exhibited the ability to metabolize endogenous and exogenous substances, especially orally administered drugs; more than 50 Food and Drug Administration (FDA)-approved drugs have been reported to have clinical interaction with gut microbiota (Wilson and Nicholson, 2017; Zhang et al., 2018). Increasing evidence showed that gut microbiota could directly affect the metabolism of drugs and alter their pharmacological effects.

In general, the oral route of drug administration is still the easiest and convenient way for drug application (Sastry et al., 2000). The liver has long been considered to be the major organ for drug metabolism, which is mainly responsible for the hydrophobic xenobiotic conversion of drugs into hydrophilic products (Kim, 2015), while the GI microflora mainly metabolize the phytochemicals into hydrophobic compounds through various microbial enzymes (Choi et al., 2018). More importantly, metabolizing drugs through the gut microbiota prior to absorption can alter the systemic bioavailability of certain drugs. Most herbal medicines are extracted by water or ethanol to generate decorations, infusions, or tincture for oral administration (Xu et al., 2017). A wide range of herbal medicine components, including ginsenosides from *Ginseng* (Kim, 2018), astilbin from *Smilax glabra* Roxb (Zhao et al., 2014), and berberine from *Coptis chinensis* (Feng R. et al., 2015), inevitably come into the GI tract. Orally administered herbal medicines are metabolized by the gut microbial enzyme before their absorption into the blood. However, studies on the impact of the gut microbial on drug metabolism and efficacy are still insufficient.

Glycyrrhizic acid (GL) is the major bioactive triterpene glycoside of the Chinese herb gancao, which has a variety of biological activities, such as anti-inflammatory, antioxidation, antiviral, immunoregulation, and liver protection (Armanini et al., 2002; Hosseinzadeh and Nassiri-Asl, 2015; Han et al., 2017). However, GL is difficult to absorb after oral administration because of its large relative molecular mass and polarity. GL needs to be deglycosylated into glycyrrhetinic acid (GA) by various gut microbiota, which is then absorbed into the blood to show efficacy (Akao, 2000). Additionally, our previous research work showed that the relative abundance of *Lactobacillus* was extremely decreased in the liver cirrhosis group compared with the healthy group. Accordingly, it was speculated that GL bioavailability was influenced by a shortage of *Lactobacillus* under a pathological state (Yuan et al., 2019). Recent research work showed that the bioavailability of orally administered drugs was directly influenced by the disturbance of gut microbiota. *Escherichia coli* Nissle 1917 could lead to a 43% increase of amiodarone area under the curve (AUC) compared with that in the control samples (Matuskova et al., 2014), while *Lactobacillus reuteri* K8 could decrease orally administered acetaminophen to 68.4% compared with that in the normal control group (Kim et al., 2018). Nevertheless, the knowledge of the effects of administration of *Lactobacillus* on GL pharmacokinetics has not been investigated, especially under a pathological state, which may provide new strategies to improve the therapeutic efficacy of GL.

Therefore, in this current study, three lactobacilli for GL biotransformation were firstly screened. *Lactobacillus murinus* was isolated from healthy rats’ feces and identified by 16S rDNA sequencing. *L. murinus* was considered as the predominant *Lactobacillus* species in rat gut, one of the most frequently found *Lactobacillus* strains. *Lactobacillus acidophilus* and *Lactobacillus rhamnosus* were both reported as beneficial probiotics, and *L. acidophilus* has been proven to play an important role in the deglucosylation step of glycoconjugated phytochemicals (Theilmann et al., 2017). Meanwhile, *L. rhamnosus* was demonstrated to have the ability for GL bioconversion in our previous research work. Therefore, the two common and available lactobacilli *L. acidophilus* and *L. rhamnosus* were chosen for the GL bioconversion compared with *L. murinus* in our study. Due to having the highest GL biotransformation rate as well as itself originating from the rat gut, *L. murinus* was selected for further research. Additionally, the beneficial properties of *L. murinus* on GL bioabsorption were primarily investigated *in vitro*. Moreover, the potential effects of selected *L. murinus* on GL bioavailability of healthy and liver cirrhosis rat models were explored.

## Materials and Methods

### Chemicals and Reagents

GL (≥98% purity) and GA (≥98% purity) were purchased from Yuanye Bio-Technology Co. (Shanghai, China). Acetonitrile and methanol were purchased from Merck KGaA (Darmstadt, Germany). Formic acid (FA) was bought from ACS (Wilmington, United States), and distilled water was prepared using a Milli-Q purification system (Millipore, Billerica, MA, United States). CCl_4_ and olive oil were purchased from Macklin Biochemical Technology Co. (Shanghai, China). An alanine aminotransferase (ALT) assay kit and aspartate aminotransferase (AST) assay kit were purchased from Jiancheng Bioengineering Institute (Nanjing, China). A MiniBEST Universal RNA Extraction Kit, PrimeScript^TM^ RT Reagent Kit with gDNA Eraser (Perfect Real Time), and SYBR^®^ Premix Ex Taq^TM^ (Tli RNaseH Plus) were purchased from Takara Bio (Tokyo, Japan). All mobile-phase solvents were of HPLC grade, and all reagents were of analytical reagent grade.

### Animals

Specific pathogen-free Sprague-Dawley rats (180–220 g) were purchased from the Experimental Animal Center of Zhejiang Province (Zhejiang, China), housed under controlled condition (light/dark, every 12 h; temperature, 20–22°C; and humidity, 50 ± 10%) and acclimated for 1 week before the experiments. Rats were fed standard laboratory chow and water *ad libitum*. The protocols for animal studies were performed according to the National Institutes of Health Guide for the Care and Use of Laboratory Animals and were approved by the Animal Studies Ethics Committee of Nanjing University of Chinese Medicine.

### Bacterial Strains and Culture Condition

*L. rhamnosus* and *L. acidophilus* were purchased from the China General Microbiological Culture Collection Center (CGMCC) under accession numbers 1.8882 and 1.1878. *L. murinus* was isolated from healthy rats’ feces. All cultures were grown in Man, Rogosa, and Sharpe (MRS) broth (Solarbio, Beijing, China) and propagated twice in MRS broth prior to use. All strains were grown overnight at 37°C in an anaerobic culture tank (volume: 2.5 L, Mitsubishi, Tokyo, Japan) with an anaerobic gas bag.

### Caco-2 Cell Culture

Caco-2 cells were preserved in our own laboratory. Caco-2 cells were maintained at 37°C under atmospheric conditions with 5% CO_2_. Cells were cultured in Dulbecco’s modified Eagle medium (DMEM, catalog number: SH30022.01; HyClone) supplemented with 10% heat-inactivated fetal bovine serum (Gibco), 1% MEM non-essential amino acids (catalog number: 321-011-EL; Wisent), and 1% penicillin–streptomycin (catalog number: SV30010.01; HyClone). The medium was changed every 3 days. When the cells reached 80–90% confluence, the cells were collected through trypsin digestion with 0.25% trypsin–EDTA (Yeasen, Shanghai, China).

### LC–MS/MS Methods

LC–MS/MS analyses were performed using a Waters HPLC system (Milford, MA, United States) coupled to a Waters Xevo TQD triple-quadrupole mass spectrometer (Milford, MA, United States). The mass spectrometer was operated using an electrospray atmospheric pressure ionization source in negative ion mode (ESI-) with multiple reaction monitoring (MRM). The analytical column was an ACQUITY UPLC^®^ BEH C18, 2.1 × 100 mm, 1.7 μM (Milford, MA, United States), and column temperature was maintained at 40°C. The mobile phase consisted of (A) 0.4% FA in distilled water and (B) acetonitrile. A gradient program was used as follows: 0–1 min, 70–20% A; 1–3 min, 20% A; 3–3.5 min, 20–70% A; 3.5–4.5 min, 70% A. The flow rate was 0. ml/min, and the sample injection volume was 10 μl.

Sensitivity of MRM was optimized by infusing a mixture of GL and GA, 1 μg/ml each, in the mobile phase. The capillary voltage was maintained at 2.66 kV, and the cone was set to 60 V. The desolvation and ion source temperatures were 300 and 150°C, respectively. The drying gas flow was set at 50 L/min. To assay all analytes, both quadrupoles were maintained at unit resolution, and the transitions (precursor to daughter) monitored were *m*/*z* 821.3 → 851 for GL and *m*/*z* 469.1 → 470 for GA. MRM data were acquired, and the chromatograms were integrated using a MassLynx V4.1 workstation.

### Transwell Experimentation and Sample Preparation

Cells were seeded onto 12-well Transwell plates (0.4-μM-pore-size polyester membrane insert, catalog number: 3460; Corning) at a concentration of 1.5 × 10^5^ cells/cm^2^. Cells were cultured for 21 days as previously described, and the medium was replaced every 2 days. Transepithelial electric resistance (TEER) assays were undertaken to confirm Caco-2 monolayer integrity. The cell monolayers were considered tight enough for the transport experiments when the TEER value was above 300 Ω⋅cm^2^. Prior to absorption experimentation, apical and basolateral compartments were washed three times and incubated in Hanks’ balanced salt solution (catalog number: RNBF9191; Sigma) at 37°C. Cells were treated apically with 1,000 μM GL or 10 μM GA in the presence or absence of 10^9^ colony-forming unit (CFU)/ml of *L. murinus*. Basolateral sampling (100 μl) with replacement was performed at 2 h.

The samples were diluted with methanol and vortex-mixed for 5 min and then centrifuged at 14,000 rpm for 7 min. The supernatant was injected into the LC–MS/MS analysis system. Apparent permeability coefficients (*P*_app_) were calculated using the equation following:

$$P_{\text{app}}\,\left(\mathrm{cm}/s\right)=\left(\text{d}\,Q/\text{d}\,t\right)\times \left(1/C\,A_{0}\right)$$

where d*Q*/d*t* is the transport rate on the receiver side (μg/s), *A* is the membrane surface area of the inset (1.1 cm^2^), and *C*_0_ is the initial drug concentration in the donor compartment (μg/ml).

### Caco-2 and Bacterial Cell Co-culture

*L. murinus* from stationary-phase cultures were washed by phosphate-buffered saline (PBS), following centrifugation at 2,500 × *g* for 5 min and resuspension in DMEM. Approximately 10^9^ CFUs were added to each well. Caco-2 cells cultured in 12-well plates (Corning) were prepared 24 h prior to the adhesion assay. The cultures were washed three times by PBS, replenished with serum- and antibiotic-free medium (DMEM) to eliminate any interference, and incubated for 24 h. On the day of the assay, the co-cultures were washed four times with PBS, a bacteria-supplemented medium was added to each well, and plates were incubated at 37°C in a 5% CO_2_ atmosphere for 3 h.

### Scanning Electron Microscopy

The Caco-2 monolayers cells were grown and maintained in a method similar to that mentioned above in 12-well tissue culture plates. After the incubation of bacteria with Caco-2 cells for 3 h, the cell monolayer with adhered bacteria was washed four times with PBS to remove the unbound bacteria. Later on, they were fixed with 2.5% glutaraldehyde and incubated at 4°C overnight. Alcohol dehydration in ascending gradation was performed, and the slides were coated with gold plating and observed under a scanning electron microscopy (SEM) (Hitachi SU8010, Japan).

### RNA Extraction and Quantitative Real-Time PCR

Total RNA was isolated from Caco-2 samples using a MiniBEST Universal RNA Extraction Kit and reverse-transcribed into cDNA using a PrimeScript^TM^ RT Reagent Kit with gDNA Eraser according to the manufacturer’s protocol. RNA and cDNA extracts were stored until use at −80 and −20°C, respectively. Custom primers were listed in Supplementary Table 1. Primers were characterized by melting curve analysis, agarose gel electrophoresis, and DNA sequencing and synthesized at GenScript Co. (Nanjing, China). Quantitative RT-PCR was performed on a CFX96 real-time PCR detection system (Bio-Rad, United States) using SYBR^®^ Premix Ex Taq^TM^ (Tli RNaseH Plus), Bulk. The relative expression of each gene was measured in triplicate via the comparative cycle threshold method, and we used the housekeeping gene *GAPDH* as the internal standard.

### Experimental Design and Model of Liver Fibrosis

We designed the following liver fibrosis experimental protocol as shown in Figure 4A. Rats were randomized into four groups (*n* = 6 for each group) as follows: the control group treated with normal saline (control group), supplementation with *L. murinus* (control + *L. murinus* group), the CCl_4_-induced fibrosis group treated with normal saline (NS group), and supplementation with *L. murinus* (NS + *L. murinus* group). Liver fibrosis was induced via an intraperitoneal injection of a 50% (V/V) CCl_4_ solution in olive oil twice per week at a dose of 1 ml/kg for 6 weeks. In the control rats, CCl_4_ was replaced with olive oil (Shi et al., 2017). At the seventh week of the experiment, animals in the NS and control groups were treated with normal saline at a dose of 1 ml, and animals in other groups were treated with a dose of 1 ml *L. murinus* by gavage once daily for 1 week. Blood was taken from the orbital vein, and blood samples were centrifuged at 3,000 × *g* for 15 min at room temperature to separate the serum for analysis. The serum levels of ALT and AST were assessed using an assay kit according to the manufacturer’s directions.

### Pharmacokinetic Experiments

Rats pretreated with *L. murinus* or normal saline (*n* = 6) were administered an oral (10 mg/kg) dose of GL dissolved in 0.5% sodium carboxymethyl cellulose (CMC-Na) (5 mg/ml) 24 h after completing the seven consecutive daily treatment with physiological saline (control) or *L. murinus* dissolved in physiological saline (1 × 10^9^ CFU/mouse). Whole-blood samples were taken from the orbital vein into tubes with heparin sodium at 0.17, 0.33, 0.5, 0.75, 1, 2, 4, 6, 8, 12, 24, 48, 72 h after oral administration (Zhou et al., 2013; Wang et al., 2017). Plasma was harvested by centrifugation at 8,000 rpm for 10 min and stored at −20°C until analyzed.

### Blood Sample Preparation

A 100 μl plasma sample was deproteinized with 1,000 μl methanol containing 10 μl internal standard (2 μg/ml). The sample was vortex-mixed for 5 min and then centrifuged at 14,000 rpm for 7 min. The supernatant was evaporated to dryness under nitrogen gas and redissolved in 80 μl mobile phase. After centrifugation at 14,000 rpm for 7 min, an aliquot of 10 μl of each sample was injected into the LC–MS/MS analysis system.

### Pharmacokinetic Analysis

After analyzing plasma samples, we obtained the plasma concentration–time profiles. The maximum plasma concentration (*C*_max_) and the time taken to reach max (*T*_max_) for GL or GA were estimated directly from the plasma concentration–time profiles. The Drug and Statistics v3.0 (Drug Clinical Research Center of Shanghai University of Traditional Chinese Medicine, Shanghai, China) was used with a non-compartmental statistical model to determine other kinetic parameters of the plasma samples.

### Statistical Analysis

GraphPad Prism 5 software (GraphPad Software, San Diego, CA, United States) was used to perform statistical analysis. The data were presented as mean ± standard derivation if normally distributed, and statistical significance was analyzed by Student’s *t*-test or ANOVA. Otherwise, the values were presented as median (25th and 75th) and analyzed by a non-parametric test (Mann–Whitney test). *P* < 0.05 was considered as statistically significant.

## Results

### *In vitro* Screening of *Lactobacillus* for GL Biotransformation

**FIGURE 1:**
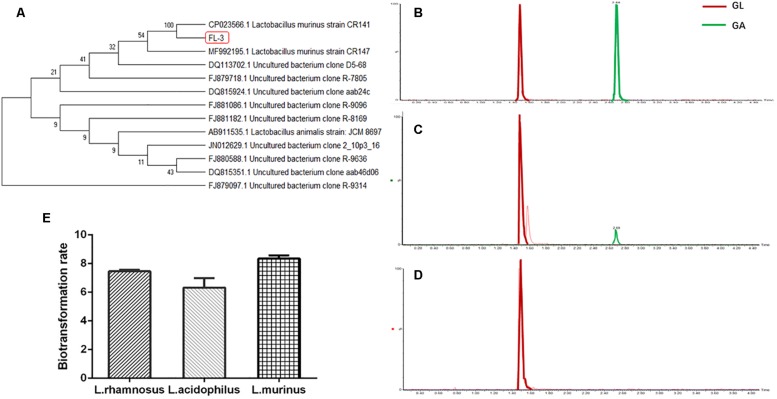
*In vitro* screening of *Lactobacillus* for GL biotransformation. **(A)** Phylogenetic relationships of the *Lactobacillus* isolated from healthy rats’ feces based on 16S rDNA gene sequencing. The tree was constructed using the neighbor-joining method in MEGA X. **(B)** UPLC–MS/MS analysis of GL and GA standard; extracted chromatograms of standard GA [M-H]^–^ = 469; extracted chromatograms of standard GL [M-H]^–^ = 821. **(C)** GL incubated with *L. murinus.* **(D)** GL incubated without *L. murinus.* **(E)** Biotransformation rate of GL screened by *L. murinus*, *L. rhamnosus*, and *L. acidophilus in vitro*.

*Lactobacillus* is largely considered to play a beneficial role for the host’s health; moreover, in our previous research work, *Lactobacillus* was significantly reduced in the pathological condition of rat liver cirrhosis, which was speculated to have an effect on GL bioavailability. In order to investigate the relationship between *Lactobacillus* and GL bioabsorption, we firstly sought to isolate the *Lactobacillus* from healthy rats’ feces on an MRS medium. The strain was identified as *L. murinus* by 16S rDNA sequencing in Figure 1A. Next, we cultured *L. murinus* and two other common and available lactobacilli, *L. rhamnosus* and *L. acidophilus*, with GL *in vitro* and screened for the GL biotransformation ability. All three *Lactobacillus* species can bioconvert the GL to GA, and the UPLC/MS results of GL biotransformation by *L. murinus* were analyzed as the representative for the other two lactobacilli. Our results showed that GL could be partially bioconverted to GA *in vitro* by *L. murinus* (Figures 1B,C), and there are no GA produced without the addition of *L. murinus*,which demonstrated that GL could not convert to GA spontaneously (Figure 1D). Among the three lactobacilli, *L. murinus* has the highest transformation rate, about 8.3% in 24 h culture, compared with the other two *Lactobacillus* strains (Figure 1E). *L. murinus* was selected for further research.

### *L. murinus* Promoted the Translocation of GL on the Caco-2 Transwell Model

**FIGURE 2:**
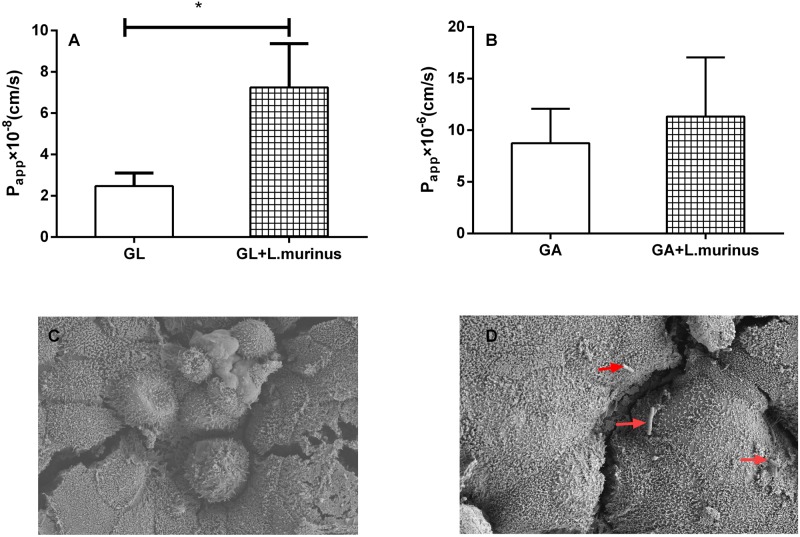
*L. murinus* promotes the translocation of GL on the Caco-2 Transwell model. **(A)** The effect of *L. murinus* on the *P*_app_ values of GL. **(B)** The effect of *L. murinus* on the *P*_app_ values of GA. **(C)** SEM images of the Caco-2 cells as control. **(D)** SEM images of *L. murinus* adherence to the Caco-2. Data are represented as the mean ± SD (*n* = 3). *P < 0.05 compared with the control group.

Furthermore, the ability of *L. murinus* to mediate the absorption of GL and GA was investigated; we used the Caco-2 Transwell model of the small intense *in vitro* to investigate the potential effect on GL intestinal absorption by *L. murinus*. As shown in Figure 2A, *L. murinus* could significantly increase the Caco-2 apical–basolateral translocation of GL compared to un-supplemented controls. Similar to that of GL, the absorption of GA was moderately increased compared with the control group (Figure 2B). These findings support the idea that *L. murinus* potentially promotes the absorption of GL and GA.

### Adherence of *L. murinus* to the Caco-2 Cells

Adherence to intestinal surfaces is usually considered to be a defining activity of a probiotic. *Lactobacillus* was reported to possess mucin-binding proteins, which are known as one of the effector molecules involved in its adherence to the host (Kaushik et al., 2009). In our present study, SEM analysis of *L. murinus* adherence to Caco-2 cells was performed. Our results showed that *L. murinus* definitely bind to the surface of the cell line (Figures 2C,D), which suggests that *L. murinus* could have direct interaction with the GI cell line.

### *L. murinus* Decreased the Expression of Transporter Genes on Caco-2 Cells

**FIGURE 3:**
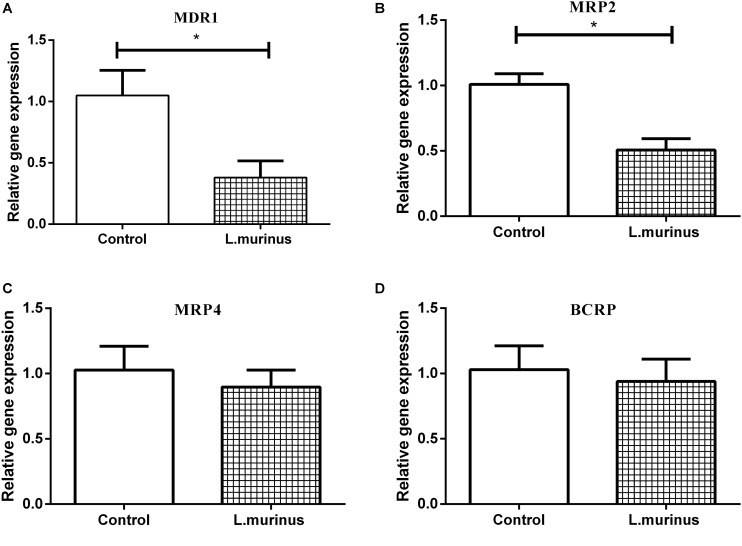
Effect of *L. murinus* on relative gene expression of transporter genes on Caco-2 cells. Only addition with GL as the control group. **(A)** The gene expression of MDR1 treated with *L. murinus*. **(B)** The gene expression of MRP2 treated with *L. murinus*. **(C)** The gene expression of MRP4 treated with *L. murinus*. **(D)** The gene expression of BCRP treated with *L. murinus.* Data are represented as the mean ± SD (*n* = 3). *P < 0.05 compared with the control group.

The expression of transporter genes such as P-glycoprotein, multidrug resistance protein 1/2 (MRP1/MRP2), and breast cancer resistance protein (BCRP) plays an important role in the absorption of many drugs. The adhesion of *L. murinus* to Caco-2 cells was supposed to have induced a variation in the mRNA expression of transporter genes. Caco-2 cells are cultured as a monolayer and spontaneously differentiate into enterocytes, which can form tight junctions between cells and express transporters. Four different transporter gene expressions on Caco-2 cells were investigated by co-culturing with *L. murinus*. The results showed that the addition of *L. murinus* to Caco-2 cells could remarkably downregulate the gene expression of MDR1 and MRP2 compared to untreated cultures (Figures 3A,B). whereas the gene expression levels of MRP4 and BCRP were not significantly changed (Figures 3C,D). Our results indicated that the downregulation of transporter genes may have contributed to the absorption of GL and GA *in vivo*.

### GL Oral Bioavailability Decreased Significantly in Liver Injury Rats

**FIGURE 4:**
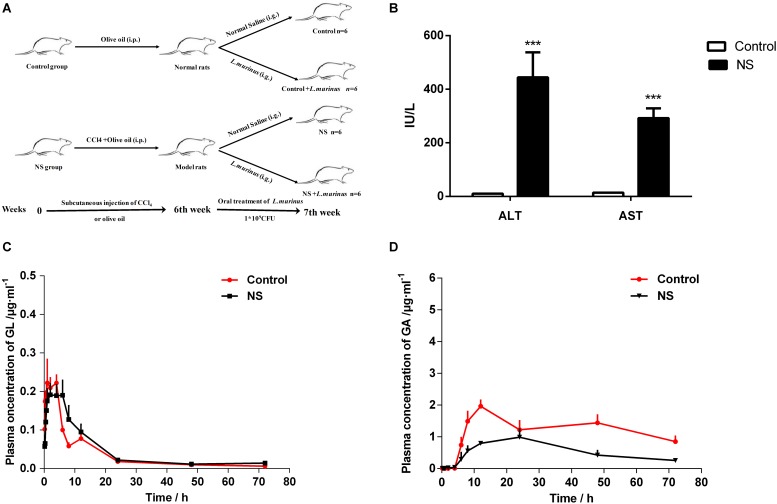
Bioavailability of GL was decreased by liver cirrhosis. **(A)** Experimental protocol of control group and liver fibrosis model group. **(B)** Rat serum levels of ALT and AST. **(C)** Plasma concentration-time curves of GL in healthy and liver cirrhosis group. **(D)** Plasma concentration–time curves of GA in healthy and liver cirrhosis group. Data are represented as the mean ± S.D. (*n* = 6). ***P < 0.001 compared with control group.

CCl_4_ was used to induce liver cirrhosis in rats; the biochemical analysis of ALT and AST revealed that CCl_4_ treatment induced a liver injury in the model group (Figure 4B). The plasma concentration levels of GL and GA were determined after GL oral administration. The mean plasma concentration–time profiles of GL and GA in the control and model groups are shown in Figures 4C,D, and the resultant pharmacokinetic data were shown in Table 1. The *C*_max_ values of GL in the control group and model group were 0.29 ± 0.11 and 0.24 ± 0.08 μg/ml, respectively, while the *C*_max_ values of GA in the control group and model group were 2.05 ± 0.48 and 1.16 ± 0.42 μg/ml, respectively. Meanwhile, the AUC value was also significantly decreased in the model group (39.83 ± 20.49 μg/ml h) compared with that in the control group (88.57 ± 25.81 μg/ml h).

**TABLE 1:** Pharmacokinetic parameters of GL and GA after oral administration in rats.

**Pharmacokinetic parameters**	**GL**			**GA**		
	**Control**	**NS**	**NS + *L. murinus***	**Control**	**NS**	**NS + *L. murinus***
*T*_max_ (h)	1.721.29	4.9584.149	2.142.93	17.3315.11	18.006.57	28.0016.40
*C*_max_ (μg/ml)	0.290.11	0.240.08	0.600.48*	2.050.48	1.160.42**	2.080.49**
AUC (0–*t*) (μg/ml h)	2.660.56	3.220.86	6.110.71***	88.5725.81	39.8320.49**	96.2723.70**
CLz/F/L⋅(h kg)^–1^	17.022.72	15.6423.309	7.150.99***	0.350.24	1.521.08*	0.370.18*

Control, healthy group; NS, liver cirrhosis group; NS + *L. murinus*, liver cirrhosis group supplementation with *L. murinus*. Data are represented as the mean ± SD (*n* = 6). Compared with the control group, * *P* < 0.05, ** *P* < 0.01, *** *P* < 0.001.

It has been reported that GL is hardly absorbed through oral administration. Our results showed that the plasma concentration of GL is quite low and that there is no difference between the two groups. However, the concentration of GA metabolized from GL was significantly decreased in the model group. These data indicated that the dysbacteriosis caused by liver injury directly influenced the bioavailability of GL.

### *L. murinus* Improved the Bioavailability of GL Under a Pathological State

**FIGURE 5:**
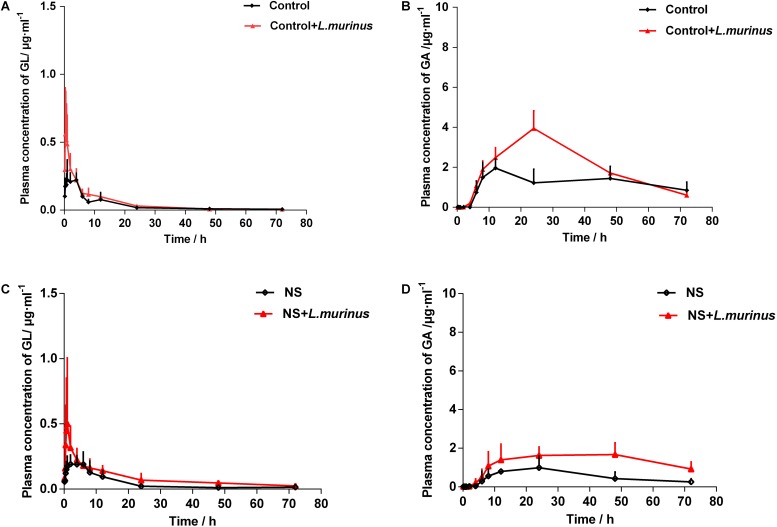
*L. murinus* promoted the bioavailability of GL under a pathological state. **(A)** Plasma concentration profiles of GL treated with *L. murinus* in the healthy group. **(B)** Plasma concentration profiles of GA treated with *L. murinus* in the healthy group. **(C)** Plasma concentration profiles of GL treated with *L. murinus* in the model group. **(D)** Plasma concentration profiles of GA treated with *L. murinus* in the model group. Data are represented as the mean ± SD (*n* = 6).

To further confirm whether the pharmacokinetics of GL was influenced by *Lactobacillus in vivo*, *L. murinus* was selected to be orally administered into the healthy and model groups by a daily gavage protocol. The plasma concentration levels of GL and GA were determined after the oral administration of *L. murinus*. The plasma concentration-versus-time curves for GL and GA are shown in Figure 5. The AUC value of GL was slightly increased to 3.80 ± 2.49 μg/ml h compared with that in the control group at 2.66 ± 0.56 μg/ml h (Figure 5A), while the AUC value of GA was significantly increased to 147.70 ± 58.62 μg/ml h compared with the control group 88.57 ± 25.81 μg/ml h (Figure 5B).

As mentioned above, the bioavailability of GL was remarkably decreased in the rats with liver injury. The addition of *L. murinus* in the model rats was investigated, and the resultant pharmacokinetic parameters were described in Table 1. Remarkably increased plasma levels of GL (AUC = 6.11 ± 0.71 μg/ml h) and GA (AUC = 96.27 ± 23.70 μg/ml h) were observed after *L. murinus* administration to rats in comparison with the samples from the model group with a GL AUC value of 3.22 ± 0.86 μg/ml h and GA AUC value of 39.83 ± 20.49 μg/ml h (Figures 5C,D). Not only did application of *L. murinus* increase the bioabsorption of GL in the normal condition, more importantly, it could significantly promote the bioavailability of GL in the liver cirrhosis models. Meanwhile, these results supported the role of gut microbiota, especially *Lactobacillus*, in GL bioabsorption.

## Discussion

GL, isolated from the traditional Chinese medicine liquorice, is known for its detoxifying and hepatoprotective properties, as well as many other pharmacological activities (Li et al., 2014). Orally administered GL is deglycosylated to GA by gut microbiota, which is then absorbed into the blood to have a therapeutic effect. Therefore, the pharmacokinetic parameters of oral GL are hugely influenced by the fluctuating gut composition, especially in certain pathological conditions of the GI tract that might alter the gut microbial composition.

However, the current concept of bioavailability of oral drugs is usually based on the normal animal gut (Enright et al., 2016). Emerging evidences have shown that the modification of gut microbiota can drastically alter drug clinical effectiveness (Hitchings and Kelly, 2019). Recently, *E. coli* infection was observed to significantly reduce the absorption of orally administered enrofloxacin in broilers (Guo et al., 2014). Yan Wang reported that berberine treatment lowered blood lipids and glucose effectively only in the high-fat diet (HFD)-fed hamsters with increased levels of nitrate reductase (NR) activity in the gut microbiota (Wang et al., 2017). In our previous research work, liver injury affects the gut microbiome in rats, particularly by depleting the *Lactobacillus*, which is directly involved in the bioconversion of the GL. The suppression of *Lactobacillus* by liver injury prompted us to investigate whether supplementation of *Lactobacillus* improves the bioavailability of GL, especially under a pathological state.

In the current work, we isolated the beneficial bacterium from healthy rats’ feces, which was identified as *L. murinus*. Two other common *Lactobacillus* strains, namely, *L. rhamnosus* and *L. acidophilus*, were purchased from the CGMCC as a representative and screened for GL biotransformation ability *in vitro*; our result showed that all three *Lactobacillus* species can bioconvert GL to GA, which partially demonstrated the important role of *Lactobacillus* in GL bioabsorption. The Caco-2 cell model is a standard tool to predict *in vivo* intestinal absorption of various drugs (Sambuy et al., 2005). The transfer capacity of *L. murinus* was validated on the Caco-2 model by co-culturing with GL and GA separately. Our results showed that the supplementation of *L. murinus* could increase the transport concentration of GL and GA compared with the control group. Under *in vitro* conditions, the intestinal mucin layer was considered as a suitable model for adherence due to the presence of specific receptors (Devi et al., 2018). It was noted that lots of *Lactobacillus* species possess this functional property: mucin proteins are involved in the adherence of *L. rhamnosus* to the host (Barnett et al., 2018), and *Lactobacillus plantarum* 299v adheres to mannose residues on intestinal epithelial cells (IECs) (Pretzer et al., 2005). *L. murinus* was also found to bind with the surface of the Caco-2 cell, suggesting its role in the interaction to the host epithelial layer.

The possible mechanism of GL transfer promotion by the addition of *L. murinus* was further investigated. The expression of intestinal transporters that are involved in drug transport plays an important role in drug bioavailability. MDR1, MRP2/MRP4, and BCRP all belong to the ABC transporters, mainly distributed in the apical membrane of IECs (Harwood et al., 2013; Wang et al., 2019). *L. murinus* treatment significantly reduced the gene expression of MDR1 and MRP2, which were directly involved in drug efflux, while MRP4 and BCRP were not affected by co-culturing with *L. murinus*. GL was reported to be a substrate of P-gp, which can reduce the absorption of GL (Feng X. C. et al., 2015). However, P-gp expression and function under normal and inflammatory conditions were found to be significantly enhanced by *Lactobacillus* (Saksena et al., 2011). This opposite conclusion may result from the fact that each *Lactobacillus* species has its own specific properties. Our results showed that *L. murinus* could bind to the surface of Caco-2 cells and downregulate the transporter gene expression compared with control. However, the detailed mechanism was not investigated in this study, so further research is required to address this issue.

Our results are in agreement with the speculation in our previous studies, where rats with liver cirrhosis were observed to have a significant decrease in the absorption of GL. A single dose of GL was orally administered, and the determination of blood concentration of GL and GA showed that the *C*_max_ of GA in rats with liver cirrhosis was about 76.7% lower than that in normal rats. Meanwhile, the AUC measurements of GA showed a similar pattern of *C*_max_. However, the C_max_ and AUC values of GL were found to have no significant changes between the two groups, probably due to the oral administration of GL being hard to be directly absorbed into the blood whether under a healthy or pathological state. Furthermore, our results revealed that the supplementation of *L. murinus* promoted the bioabsorption of GL in healthy rats; more importantly, the blood concentrations of GL and GA in rats with liver cirrhosis were increased significantly under the treatment of *L. murinus*. A previous study has reported that the administration of *Lactobacillus* directly contributes to the improvement of liver injury (Shi et al., 2017). Supplementation of *L. murinus* could regulate the imbalance of gut microbiota caused by liver cirrhosis; more importantly, it may have directly interacted with the intense layer and promoted GL bioavailability. Our results confirmed that the supplementation of the selected probiotic during the short term is enough to improve the pharmacokinetic characteristics of GL, especially under pathological conditions.

Probiotic intervention is gaining more attention due to its potential to confer a health benefit on the host (Salazar et al., 2019). *Lactobacillus* is one of the genera that are most commonly used as probiotics, which has exerted various beneficial functions on the host (Druart et al., 2014). *L. rhamnosus* GG was observed to ameliorate dextran sulfate sodium (DSS)- and oxazolone-induced colitis through the activation of the epidermal growth factor receptor (EGFR) (Yan et al., 2011); the administration of *L. rhamnosus* LB102 reduced diet-induced obesity and inflammation, concomitant with the improvement of glucose tolerance and insulin sensitivity (Le Barz et al., 2019). *L. murinus* has also been reported to successfully colonize in rodent intestines and to possess protective properties for necrotizing enterocolitis (Isani et al., 2018). And treatment of mice with *L. murinus* prevented salt-induced aggravation of actively induced experimental autoimmune encephalomyelitis and salt-sensitive hypertension (Wilck et al., 2017). Recently, *L. murinus* has been reported to significantly increase the life span and the brood size of the nematode *Caenorhabditis elegans* (Pan et al., 2018). In the current study, we display the novel aspects of the beneficial property of *Lactobacillus*, *L. murinus* was observed to have the capacity to increase the bioavailability of GL for the first time.

Growing evidences have demonstrated that gut flora disequilibrium is closely related with liver cirrhosis; in the current study, our results revealed that microbiota modification definitely influences drug absorption. Combing *in vitro* screening and *in vivo* bioabsorption experiments, our data highlighted that supplements of *L. murinus* significantly improved the bioavailability of orally administered GL, especially under pathological conditions, which may provide a novel strategy for improving the clinical therapeutic effect of liver protective drugs.

## Data Availability Statement

The datasets generated for this study can be found in the https://figshare.com/s/823ded22d8ef86a034ca.

## Ethics Statement

The animal study was reviewed and approved by Animal Studies Ethics Committee of Nanjing University of Chinese Medicine.

## Author Contributions

TY and JW carried out the sample collection and data analysis and drafted the manuscript. LC and JS revised the final draft of the manuscript. LD, who is the corresponding author, conceived of the study and helped to draft the manuscript. All authors read and approved the final manuscript.

## Conflict of Interest

The authors declare that the research was conducted in the absence of any commercial or financial relationships that could be construed as a potential conflict of interest.
