# Supplementary material for: Lactobacillus murinus Improved the Bioavailability of Orally Administered Glycyrrhizic Acid in Rats
Source: Front Microbiol. 2020 Apr 24;11:597. doi: 10.3389/fmicb.2020.00597 (PMC7193032; doi:10.3389/fmicb.2020.00597)
Supplement: Supplementary file 1 [file Table_1.DOCX]

Supplementary Material

***Lactobacillus murinus* Improved the Bioavailability**

of Orally Administered Glycyrrhizic Acid in Rats

Tianjie Yuan^a^, Jin Wang^a,b^, Letian Chen^b^, Jinjun Shan^c^, Liuqing Di^a,b^*

^1^ School of Pharmacy, Nanjing University of Chinese Medicine, Nanjing, China

^2^ Jiangsu Engineering Research Centre for Efficient Delivery System of TCM, School of Pharmacy, Nanjing University of Chinese Medicine, Nanjing, China.

^3^Jiangsu Key Laboratory of Pediatric Respiratory Disease, Nanjing University of Chinese Medicine, Nanjing, China

*** Correspondence:**Liuqing Di
diliuqing0928@163.com

# Supplementary Tables

Supplementary Table 1. Primer sequence and annealing temperature for Real-time PCR

| Gene | Forward sequence (5′–3′) | Reverse sequence (5′–3′) | Annealing temperature |
| --- | --- | --- | --- |
| GAPDH | TCCTGCACCACCAACTGCTTAG | AGTGGCAGTGATGGCATGGACT | 56.3℃ |
| MDR1 | TCTTGAAGGGCCTGAACCTG | AGTCATAGGCATTGGCTTCC | 56.3℃ |
| MRP-2 | TGAGCAAGTTTGAAACGCACAT | AGCTCTTCTCCTGCCGTCTCT | 58.3℃ |
| MRP-4 | GCTCAGGTTGCCTATGTGCT | CGGTTACATTTCCTCCTCCA | 52℃ |
| BCRP | TGCAACATGTACTGGCGAAGA | TCTTCCACAAGCCCCAGG | 56.3℃ |
